# Exploring the Shared Diagnostic Genes in IBD and Psoriasis through Bioinformatics and Experimental Assays

**DOI:** 10.7150/ijms.107018

**Published:** 2025-03-03

**Authors:** Lichun Han, Guangfu Lin, Xiaodan Lv, Bing Han, Xiaofang Xu, Yu Li, Shiquan Li, Deyi Chen, Zhixi Huang, Guangli Gu, Xiaoping Lv

**Affiliations:** 1 Department of Gastroenterology, The First Affiliated Hospital of Guangxi Medical University, Nanning 530021, China.; 2 Department of Clinical Experimental Medicine, The First Affiliated Hospital of Guangxi Medical University, Nanning 530021, China.; 3 Department of Gastroenterology, The Fourth Affiliated Hospital of Guangxi Medical University, Liuzhou 545005, China.

**Keywords:** inflammatory bowel disease, psoriasis, shared diagnostic genes, bioinformatics, machine learning

## Abstract

**Background:** Inflammatory bowel disease (IBD) is a persistent, non-specific inflammation affecting the intestines. Psoriasis is a long-lasting inflammatory disorder of the skin. There is a comorbidity correlation between IBD and psoriasis, but the specific pathogenesis of the comorbidity is unclear.

**Materials and methods:** In this study, we analyzed datasets sourced from the Gene Expression Omnibus (GEO) database, and identified shared genes of IBD and psoriasis through differential expression analysis and weighted gene co-expression network analysis (WGCNA). Then three machine learning algorithms were applied to identify shared diagnostic genes. Next, the validation of shared diagnostic genes was evaluated with ROC curves, with the AUC determined. Subsequently, single sample gene set enrichment analysis (ssGSEA) and immune infiltration analysis were conducted. Furthermore, we obtained potential drugs such as securinine in the Drug Signature Database (DsigDB) and 7 traditional Chinese medicines in the Coremine database, which might have therapeutic effects on the comorbidity of IBD and psoriasis. Finally, we confirmed the expression of the shared diagnostic gene in colitis and psoriasis mice tissues through RT-PCR, Western blot and immunohistochemistry (IHC) methods.

**Results:** The results showed that AQP9 had the highest diagnostic value for two diseases. AQP9 had AUC values of 93.681% for UC, 89.629% for CD,and 78.689% for psoriasis in the internal validation datasets. In the external validation datasets, AQP9 had AUC values of 90.394% for UC, 93.909% for CD,and 82.906% for psoriasis. Immune infiltration analysis and ssGSEA revealed that AQP9 might impact the disease process of IBD and psoriasis by participating in the NF-kappaB signaling pathway, and modulating immune cell differentiation. Furthermore, the expression levels of AQP9 were consistently validated, showing upregulation in IBD and downregulation in psoriasis, compared to the control group.

**Conclusions:** This study revealed the shared diagnostic genes and potential mechanisms of the comorbidity of IBD and psoriasis, providing new directions for future research on exploring the comorbidity mechanisms and treatment targets.

## Introduction

Inflammatory bowel disease (IBD) is a non-specific, unexplained inflammation affecting the intestines of unknown origin, including Crohn's disease (CD) and ulcerative colitis (UC). IBD is usually a lifelong concomitant disease characterized by alternating recurrence and remission. Its primary clinical symptoms are abdominal pain, diarrhea, and the release of mucus and blood in stools 1, 2. The most frequent symptoms of IBD are found in the gastrointestinal tract, but IBD is often accompanied by extraintestinal manifestations (EIMs) and comorbidities. Previous studies mainly focused on the EIMs of IBD. However, comorbidities in IBD had been often overlooked. IBD comorbidities are defined as a group of diseases that coexist with and are associated with IBD under specific conditions, including psoriasis, rheumatoid arthritis, psychological and psychiatric disorders, and cardiovascular diseases. Compared to IBD patients without comorbidities, the quality of the IBD patients with comorbidities usually showed much more impact by it 3-5.

Psoriasis is a long-term inflammatory skin disorder, with a global incidence varying from 0.1% in East Asia to 1.5% in western Europe 6. Psoriasis can be divided into several clinical forms, with plaque psoriasis being the most common. Psoriasis has a chronic and recurrent course that cannot be cured at present 7. Psoriasis is caused by changes in skin barrier function and dysregulation of skin immune response, and its pathogenesis involves genetic susceptibility, environmental factors, and immune dysregulation 6, 8. Various autoimmune diseases such as IBD had been reported to be related to psoriasis 9.

Psoriasis and IBD are recurrent, long-lasting diseases driven by immune system dysfunction. The occurrence of psoriasis among IBD patients stood at 4.2%, surpassing that of the general population. Simultaneously, the overall rate of IBD among psoriasis patients was 1.2% 9, 10. The comorbidity of psoriasis and IBD exacerbates the decline in patient quality of life and affects the treatment and prognosis of the disease. The advent of biological agents has significantly transformed the management of immune-driven inflammatory conditions, such as IBD and psoriasis. It could quickly control disease activity, promote mucosal healing, improve quality of life, and reduce surgical risk. However, paradoxical phenomenon had been reported in multiple case studies and clinical trials, such as the progression of psoriasis after using TNF inhibitors for IBD treatment 11, 12. Therefore, exploring the potential mechanism of comorbidity of IBD and psoriasis is particularly important, which can benefit early diagnosis and treatment in the future.

In our research, we employ an integrated approach of bioinformatics and laboratory experiments to explore the shared diagnostic genes and potential mechanism involved in the comorbidity of IBD and psoriasis. Our analysis results indicated that AQP9 may serve as a potential shared diagnostic gene of comorbidity of IBD and psoriasis. Meanwhile, the capacity of AQP9 to regulate the differentiation and migration of immune cells, including neutrophils, macrophages, and T cells, may correlate with the inflammatory conditions of IBD and psoriasis. Investigating the shared diagnostic gene and pathway in IBD and psoriasis can deepen our understanding of comorbidity pathogenesis and positively influence future strategies for diagnosis and treatment.

## Materials and methods

### Acquisition of public data

We obtained datasets information related to IBD and psoriasis from GEO database. We selected the GSE3365 and GSE14905 to screen for shared genes in IBD and psoriasis, while selecting the GSE75214 and GSE52471 as validation sets.

### Differential gene expression analysis

The LIMMA package in R software (version 4.3.2) facilitated the calculation of differentially expressed genes (DEGs) among the control and disease groups in UC, CD, and psoriasis datasets. Subsequently, we employed volcano plots to visualize the results of the gene expression analysis.

### WGCNA

WGCNA aimed to pinpoint gene modules for co-expression and investigate the connections between gene networks and phenotypes, including identifying key genes within the network. We utilized the 'WGCNA' R package to categorize genes into groups according to their co-expression similarities among samples. The input data, which was normalized with R-based limma algorithms, was utilized to identify the co-expression modules within the genes of UC/CD and psoriasis, and to examine the relationship between gene modules and clinical traits.

### Identifying shared genes and conducting enrichment analysis

We defined shared genes potentially linked to the pathogenesis of IBD and psoriasis comorbidity by merging intersecting DEGs with key module genes identified through WGCNA. To uncover the biological functions and pathways associated with these shared genes, we performed functional enrichment analysis using the Gene Ontology (GO) and Kyoto Encyclopedia of Genes and Genomes (KEGG) pathways via the 'clusterProfiler' package. Bubble plots were utilized to depict the enrichment analysis outcomes, where P<0.05 was considered to signify significant findings.

### Selecting features using three machine algorithms

We applied shared genes in three machine learning methods: Least Absolute Shrinkage and Selection Operator (LASSO), Support Vector Machine-Recursive Feature Elimination (SVM-RFE), and Random Forest (RF) to screen features related to the pathogenesis of IBD and psoriasis. To ensure the reproducibility of these methods, we set seeds. After filtering through these algorithms, we combined the results from UC and psoriasis, and CD and psoriasis to identify shared diagnostic genes.

### Internal and external validation of shared diagnostic genes

To assess the predictive value of the diagnostic genes identified by the machine algorithms, we performed internal validation using the training datasets, followed by external validation with external datasets. The data for the two validation datasets were obtained through the GEO database and standardized using the RMA algorithm. To visualize the expression patterns of the shared diagnostic genes across various groups, box plots were used. Subsequently, the ROC curves for these genes were plotted in UC, CD, and psoriasis, and the AUC was calculated.

### ssGSEA

Using the identified common diagnostic genes, ssGSEA analysis was conducted on two training datasets with the 'clusterProfiler' package. This analysis helped explore the biological pathways in both the disease groups and control groups. Enrichment plots were employed to illustrate the key pathways shared by IBD and psoriasis, along with their NES, P, and FDR values.

### Immune infiltration analysis

We conducted immune infiltration analysis on IBD and psoriasis datasets using the CIBERSORT algorithm to estimate the relative levels of immune cells. Box plots and bar charts were utilized to compare the immune cell differences between the control and disease groups. Correlation heatmaps were created to show the relationship between the expression of shared diagnostic genes and the various differentially infiltrating immune cell types. Non-parametric Spearman correlation was employed to assess the correlation of infiltrating immune cells with shared diagnostic genes.

### Potential drug prediction

In order to explore potential therapeutic drugs for two diseases related to shared diagnostic genes, we used the DsigDB to obtain the relevant potential therapeutic drugs. After that, we chose the Coremine database to predict traditional Chinese medicine that might interact with shared diagnostic genes.

### Animal model establishment

Female Balb/c mice, aged 7-8 weeks and weighing 19-21 g, were sourced from Huachuang Xinnuo (Taizhou, China). They were given at least one week to acclimate before the experiments began. The animal study received approval from the Ethical Review Committee of the First Affiliated Hospital of Guangxi Medical University.

In this experiment, we established both control and disease groups for two diseases, with 8 mice in each group. In IBD group, we induced colitis using 2,4,6-Trinitrobenzenesulfonic acid (TNBS). Briefly, 150µl of 1%(wt/v) TNBS-olive oil-acetone solution was applied to the shaved skin for pre-sensitization, followed by the administration of 100µl of 2.5%(wt/v) TNBS in 50% ethanol into the colons of mice that had been fasted for 24 hours, 7 days after pre-sensitization. The control group was pre-sensitized with the olive oil-acetone solution and a control was administered using an equivalent volume of phosphate-buffered saline (PBS) via enema. In the psoriasis group, we induced psoriasis in mice by applying 5% imiquimod (Mingxin, Sichuan, China) to the shaved backs of mice for 8 consecutive days. The control group received an equal amount of vaseline applied to their shaved backs.

### Real-time polymerase chain reaction (RT-PCR)

To validate the expression of shared diagnostic gene identified through bioinformatics. We extracted RNA from intestinal and skin tissues using the TRIzol method and synthesized cDNA using the StarScript III All-in-one RT Mix (GenStar, Suzhou, China). RT-PCR was then performed using the All-in-one First Strand cDNA Synthesis Kit II (Seven, Beijing, China) to quantify the expression levels of AQP9 in the two diseases. Supplementary Table S1 includes the primer sequences for AQP9.

### Western blot

Total proteins were isolated from tissue specimens using RIPA Lysis Buffer, supplemented with a proteinase inhibitor (Servicebio, G2002, China). The BCA protein assay kit was utilized to quantify protein concentrations. Equal amounts of protein samples (30µg) were subjected to 12% sodium dodecyl sulfate-polyacrylamide gel electrophoresis (SDS-PAGE) and then transferred to polyvinylidene difluoride (PVDF) membranes. The membranes were blocked with 5% non-fat milk in TBST for 1 hour at room temperature and then incubated with rabbit anti-AQP9 (1:1000, Proteintech, 20380-1-AP, China) and rabbit anti-β-tubulin (1:1000, Cell Signaling Technology, 2146, USA) overnight at 4°C. The membranes were treated with secondary immunoglobulin (1:30000, Cell Signaling Technology, 5151, USA) for 1 hour at room temperature. Protein bands were observed with the assistance of an Odyssey CLx system (LI-COR, USA). Relative protein expression levels were standardized to β-tubulin and measured with Image J software.

### Immunohistochemistry

Tissue sections underwent deparaffinization and rehydration, followed by antigen retrieval using citrate buffer (pH 6.0). Sections were treated with peroxidase blocking solution for 10 minutes at room temperature to inhibit endogenous peroxidase activity, followed by three washes with PBS, each for 3 minutes. The sections were then exposed to primary antibody against AQP9 (1:500, Proteintech, 20380-1-AP, China) at 4°C overnight. After washing three times with PBS (3 min each), the sections were exposed to 100 µL of reaction enhancement reagent at 37°C for a period of 20 minutes, followed by three PBS washes, each lasting 3 minutes. Subsequently, sections were incubated with of enhanced enzyme-labeled goat anti-mouse/rabbit IgG polymer at 37°C for 20 minutes, followed by three washes with PBS, each lasting 3 minutes. For visualization, a newly made DAB chromogen solution was applied and incubated at ambient temperature for 6 minutes. After washing with tap water, sections were stained with hematoxylin for 20 seconds for counterstaining, followed by differentiation and bluing. In the final steps, the sections were dehydrated using a series of graded alcohols, cleared with xylene, and subsequently mounted with neutral balsam.

### Statistical analysis

Statistical analysis was conducted using R version 4.3.2. To compare different experimental groups, Student's t-test was employed for two-group comparisons. Data are presented as mean ± standard deviation (SD), with a significance level of P value <0.05.

## Results

### Differential genes screening

**Figure 1:**
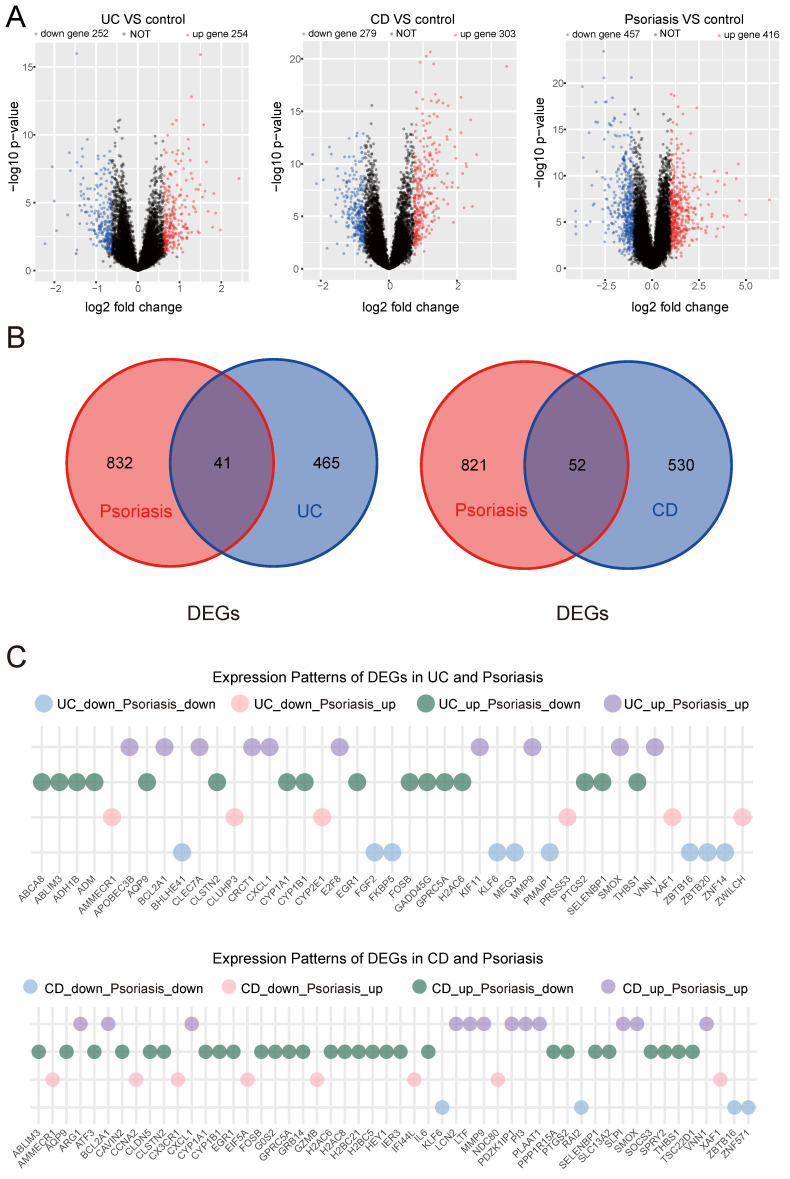
** Volcano plots and venn plots.** (A) Volcano plots of DEGs between the disease group and the control group in UC, CD and psoriasis. (B) Shared DEGs obtained through intersection of DEGs between UC/CD and psoriasis. (C) Differential expression patterns of shared DEGs between IBD and psoriasis.

Utilizing data from the open GEO database, we acquired the GSE3365 series matrix file related to IBD. The annotated GPL96 platform included 127 transcriptome datasets, comprising 42 normal, 26 UC, and 59 CD samples. DEGs between the UC/CD disease groups and control groups were identified using the limma package. For UC, we identified 506 DEGs (P value <0.05 and |log2FC|>0.618), with 254 genes upregulated and 252 downregulated. For CD, we identified 582 DEGs (P value <0.05 and |log2FC|>0.728), with 303 genes upregulated and 279 downregulated. Additionally, we downloaded the GSE14905 series matrix file related to psoriasis from the GPL570 platform, which included 82 transcriptome datasets: 21 normal and 61 psoriasis samples. In psoriasis, 873 DEGs (P value <0.05 and |log2FC|>0.989) were identified, with 416 upregulated and 457 downregulated genes. To visualize DEGs, volcano plots were utilized. Red color denoted highly expressed upregulated genes, while blue signified lowly expressed downregulated genes (Figure 1A). We intersected DEGs obtained by comparing the UC and psoriasis groups and the CD and psoriasis groups. Figure 1B showed that there was an overlap of 41 genes in the DEGs of UC and psoriasis. In the CD and psoriasis groups, a total of 52 DEGs overlap (Figure 1B).

Furthermore, we characterized the expression profiles of shared DEGs between IBD and psoriasis (Figure 1C). Comparative analysis revealed four distinct expression patterns in both UC-psoriasis and CD-psoriasis pairs: concordantly downregulated genes, concordantly upregulated genes, and genes exhibiting inverse regulation (upregulated in IBD while downregulated in psoriasis, or vice versa). These preliminary results elucidate the complex relationship between IBD and psoriasis at the transcriptional level, which may reflect potential disease-specific regulatory mechanisms underlying these chronic inflammatory conditions.

### Identification of key module genes using WGCNA

**Figure 2:**
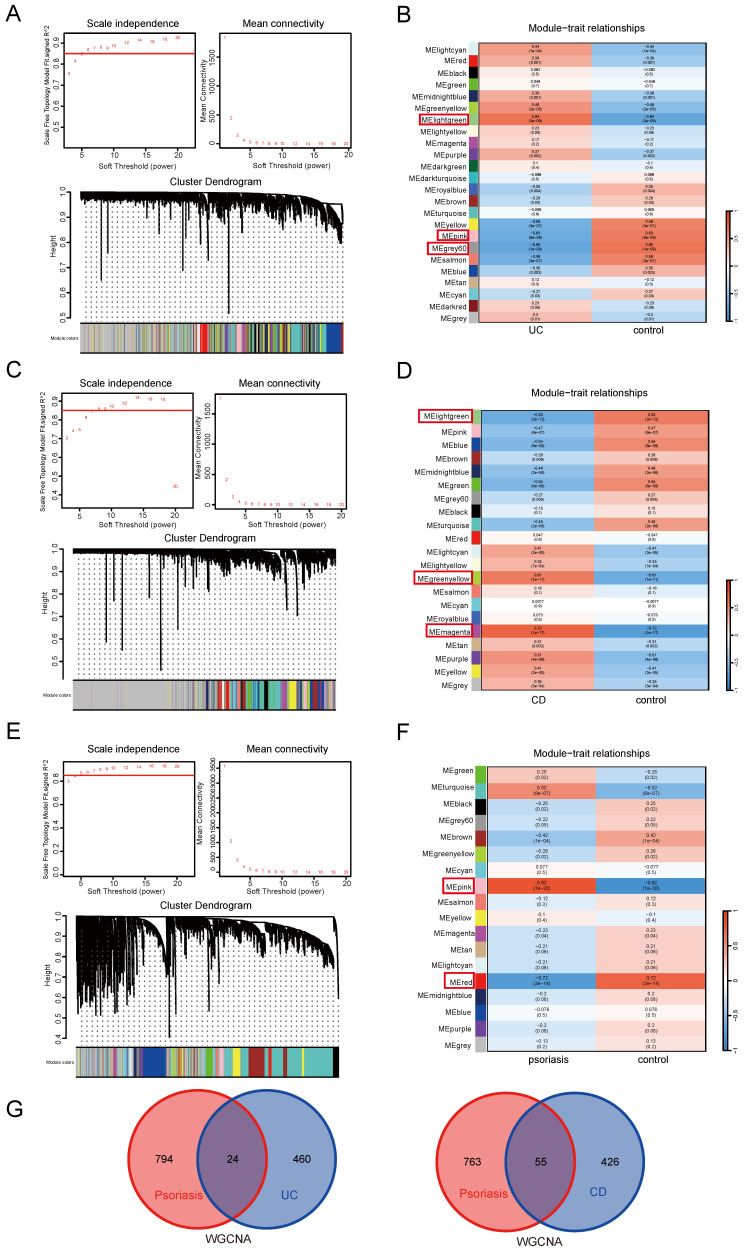
** WGCNA.** (A) Determination of soft threshold and cluster tree for UC group. (B) The correlation between modules and traits in the UC group. (C) Determination of soft threshold and cluster tree for CD group. (D) The correlation between modules and traits in the CD group. (E) Determination of soft threshold and cluster tree for psoriasis group. (F) The correlation between modules and traits in the psoriasis group. (G) Shared genes obtained from overlapping WGCNA key modules of UC/CD and psoriasis.

We employed WGCNA to investigate crucial module genes in diseases. This study utilized a soft threshold method to create a co-expression network, with the soft threshold (power value) being essential for maintaining a scale-free topology. In the UC group, to ensure a scale-free topology of the co-expression network, the fitting index was set above 0.85, and the power value was set to 6. We then built a co-expression network based on the optimal soft threshold, categorized the genes into different modules, and generated a clustering tree (Figure 2A). A total of 24 co-expression modules were identified. Modules with a significance level of P<0.05 were considered key modules. As depicted in Figure 2B, the lightgreen module showed the highest positive correlation, while the pink and grey60 modules exhibited the strongest negative correlations. These three highly correlated modules encompassed 484 genes.

WGCNA analysis was subsequently applied to the CD group, setting the fitting index above 0.85 and the power value at 8. Using the optimal soft threshold, we developed a co-expression network, categorized genes into various modules, and generated a cluster tree (Figure 2C). This analysis identified 21 co-expression modules. In Figure 2D, the magenta and greenyellow modules exhibited the strongest positive correlation, while the lightgreen module showed the strongest negative correlation. The three highly correlated key modules contained a total of 481 genes.

Meanwhile, WGCNA was applied to the psoriasis group, with the fitting index remaining above 0.85. A power value of 5 was determined to be optimal for the soft threshold (Figure 2E). We identified 18 modules, with the pink module showing significant positive correlation and the red module showing significant negative correlation (Figure 2F). These two highly correlated modules comprised a total of 818 genes. These key module genes with strong correlation might serve as important shared genes between UC, CD, and psoriasis disease groups and control groups.

We then intersected the genes screened by WGCNA obtained by comparing the UC and psoriasis groups and the CD and psoriasis groups. There were a total of 24 overlapping genes in the key module genes in WGCNA of UC and psoriasis. In the CD and psoriasis group, there were a total of 55 overlapping genes in the key module genes of WGCNA (Figure 2G).

### Shared gene screening

We define the intersection genes obtained in DEGs and WGCNA as shared genes of IBD and psoriasis. After removing one duplicate gene, 64 shared genes for UC and psoriasis were obtained. In the CD and psoriasis group, after removing 12 duplicate genes, 95 shared genes were obtained (Table S2).

### Analysis of functional enrichment in shared genes

In order to understand the common potential biological changes between IBD and psoriasis, we performed functional enrichment analysis on shared genes identified from UC-psoriasis (64 genes) and CD-psoriasis (95 genes) comparisons. The enrichment results for UC-psoriasis shared genes (Figure S1A) and CD-psoriasis shared genes (Figure S1B) revealed similar biological patterns. KEGG analysis indicated that shared genes participate in key inflammatory and immune pathways, including the IL-17, NF-kappaB, and TNF signaling pathways in IBD and psoriasis. GO functional enrichment demonstrated that these genes are involved in regulating mucosal innate immune response, humoral immune response, apoptosis signaling, and responses to lipopolysaccharides and bacterial defense. This implies that both inflammation and immune regulation play significant roles in the development of the two diseases.

### Screening shared genes for UC and psoriasis using three machine learning algorithms

**Figure 3:**
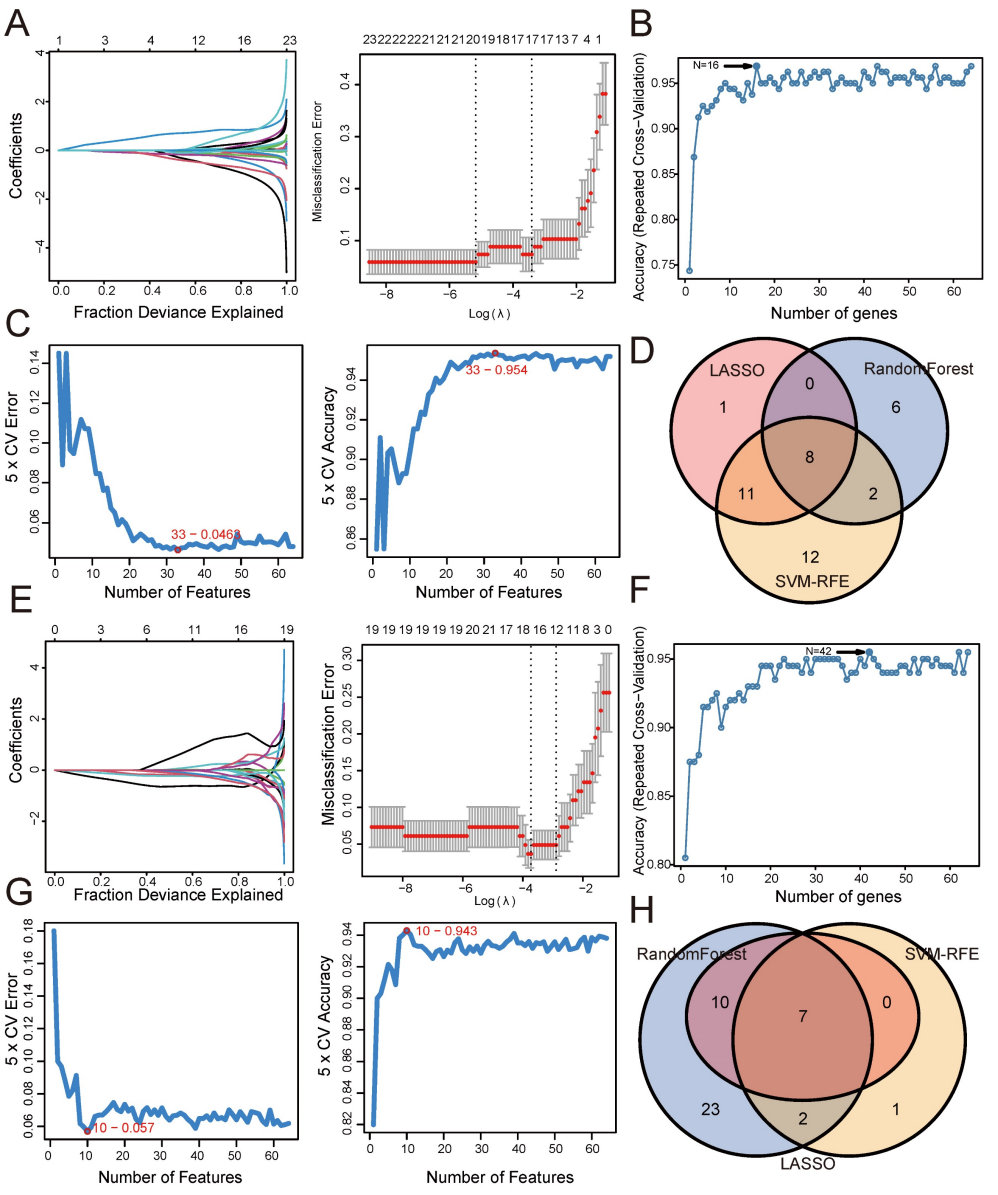
**Screening shared genes for UC and psoriasis using three machine learning algorithms.** (A) Coefficient profile plot of the LASSO model for UC. (B) Screening feature genes for UC using Random Forest algorithm. (C) Screening feature genes for UC using SVM-RFE algorithm. (D) Venn plot showed the intersection results of three algorithms in UC. (E) Coefficient profile plot of the LASSO model for psoriasis. (F) Screening feature genes for psoriasis using Random Forest algorithm. (G) Screening feature genes for psoriasis using SVM-RFE algorithm. (H) Venn plot showed the intersection results of three algorithms in psoriasis.

To further identify shared genes with significant feature values for distinguishing between disease and control groups, we utilized three machine algorithms: LASSO, SVM-RFE, and Random Forest. This screening was based on the 64 shared genes between UC and psoriasis. Figure 3 illustrates the results of applying these machine learning algorithms to UC and psoriasis. For the UC group, the LASSO coefficient distribution and optimal parameter selection chart determined a λ value of 0.005653324 (Figure 3A), identifying 20 genes with non-zero coefficients. We then inputted the 64 shared genes into the Random Forest algorithm, resulting in 16 identified genes (Figure 3B). Additionally, the SVM-RFE algorithm identified 33 genes with the lowest CV error and highest accuracy (Figure 3C). By intersecting the results from these three algorithms, we identified 8 shared biomarkers for the UC group (Figure 3D, Table 1). Similarly, for the psoriasis group, using the LASSO algorithm with a λ value of 0.02403203 identified 17 characteristic genes (Figure 3E). The Random Forest algorithm selected 42 genes (Figure 3F), and the SVM-RFE algorithm identified 10 genes (Figure 3G). The overlap of these three algorithms yielded 7 shared genes (Figure 3H, Table 1).

### Screening shared genes for CD and psoriasis using three machine learning algorithms

**Figure 4:**
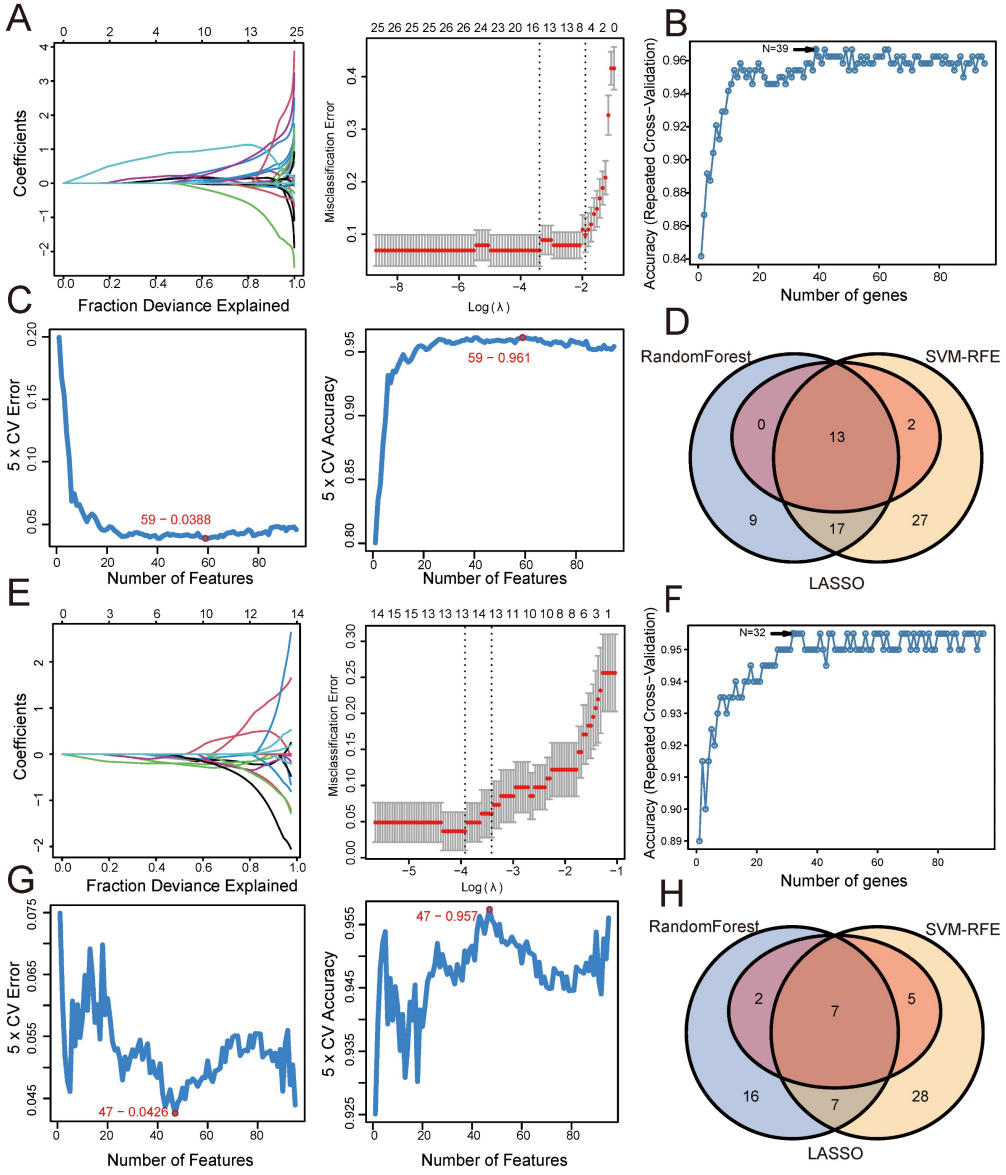
** Screening shared genes for CD and psoriasis using three machine learning algorithms.** (A) Coefficient profile plot of the LASSO model for CD. (B) Screening feature genes for CD using Random Forest algorithm. (C) Screening feature genes for CD using SVM-RFE algorithm. (D) Venn plot showed the intersection results of three algorithms in CD. (E) Coefficient profile plot of the LASSO model for psoriasis. (F) Screening feature genes for psoriasis using Random Forest algorithm. (G) Screening feature genes for psoriasis using SVM-RFE algorithm. (H) Venn plot showed the intersection results of three algorithms in psoriasis.

Similarly, based on 95 shared genes from the CD and psoriasis groups, we used three algorithms for screening. Figure 4 shows the results of applying these machine learning algorithms to CD and psoriasis. For the CD group, the LASSO coefficient distribution and the optimal parameter selection chart determined a λ value of 0.03384779 (Figure 4A), identifying 15 genes with non-zero coefficients. We inputted the shared genes into the Random Forest algorithm, resulting in 39 identified genes (Figure 4B). Additionally, the SVM-RFE algorithm identified 59 genes with the lowest CV error and highest accuracy (Figure 4C). By intersecting the results from these three algorithms, we identified 13 shared biomarkers for the CD group (Figure 4D, Table 2). Similarly, using the LASSO algorithm with a λ value of 0.01974293, the psoriasis group identified 14 characteristic genes (Figure 4E). The Random Forest algorithm selected 32 genes (Figure 4F), and the SVM-RFE algorithm identified 47 genes (Figure 4G). The overlap of these three algorithms yielded 7 shared genes in the psoriasis group (Figure 4H, Table 2).

### Validation of shared genes

**Figure 5:**
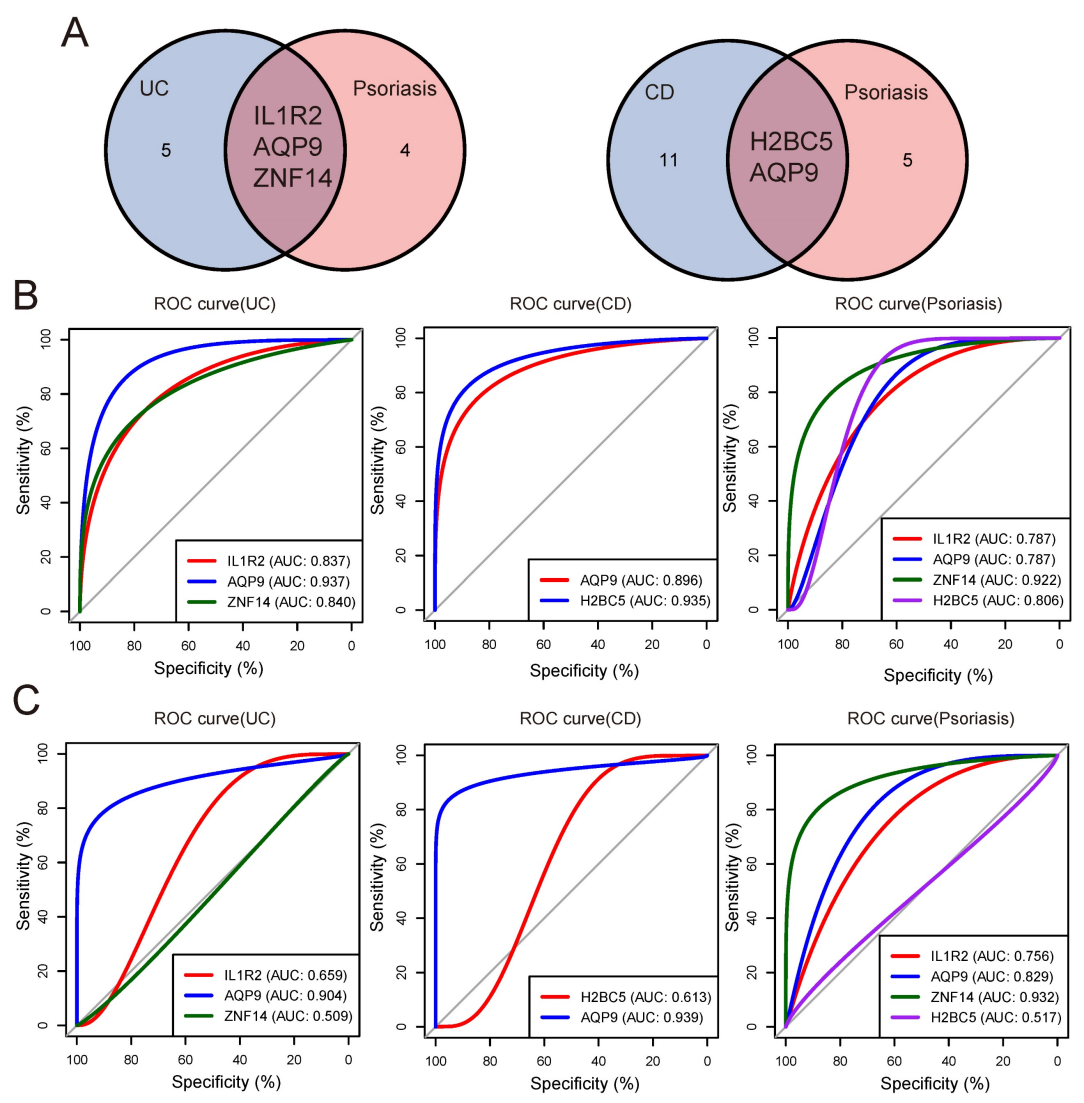
** Validation of shared diagnostic genes.** (A) The venn plot showed the shared diagnostic genes between UC/CD and psoriasis. (B) The ROC curves of shared diagnostic genes in the internal validation datasets. (C) The ROC curves of shared diagnostic genes in the external validation datasets.

In order to obtain the shared genes of IBD and psoriasis, we intersected the machine learning results of UC/CD and psoriasis groups to obtain shared genes (Figure 5A). To evaluate the predictive value of shared genes for the diagnosis of two diseases and understand their expression trends in both diseases, box plots were performed to show the expression trends of shared genes in the internal (Figure S2A) and external validation (Figure S2B). Then we plotted the ROC curve and determined the AUC. The ROC curves showed that the AUC of IL1R2, AQP9, and ZNF14 were 83.7%, 93.681%, and 83.974% in UC group, respectively. In the CD group, the AUC values of H2BC5 and AQP9 were 93.543% and 89.629%. The same ROC analysis was also performed in the psoriasis group, and the AUC of IL1R2, AQP9, ZNF14 and H2BC5 were obtained to be 78.728%, 78.698%, 92.194% and 80.562%. The predictive value of three shared genes on UC and psoriasis in the training datasets was robust (Figure 5B).

Moreover, we selected the GSE75214 dataset and GSE52471 dataset as validation sets to confirm the reliability of shared genes for IBD and psoriasis through external validation. In the UC group, the AUC of IL1R2, AQP9, and ZNF14 were 65.933%, 90.394%, and 50.914%. In the CD group, the AUC of diagnostic genes H2BC5 and AQP9 were 61.333% and 93.909%, in the psoriasis group, the AUC of IL1R2, AQP9, ZNF14 and H2BC5 were 75.641%, 82.906%, 93.162%, and 51.709% (Figure 5C).

Finally, by comparing the expression trends and diagnostic efficacy of shared genes in each group of the validation datasets, it was observed that AQP9 expression trends in the validation sets were consistent with those presented in the training datasets and had higher diagnostic value for UC/CD and psoriasis (Table 3). These findings suggest that AQP9 could serve as a shared diagnostic gene for both IBD and psoriasis.

### ssGSEA

**Figure 6:**
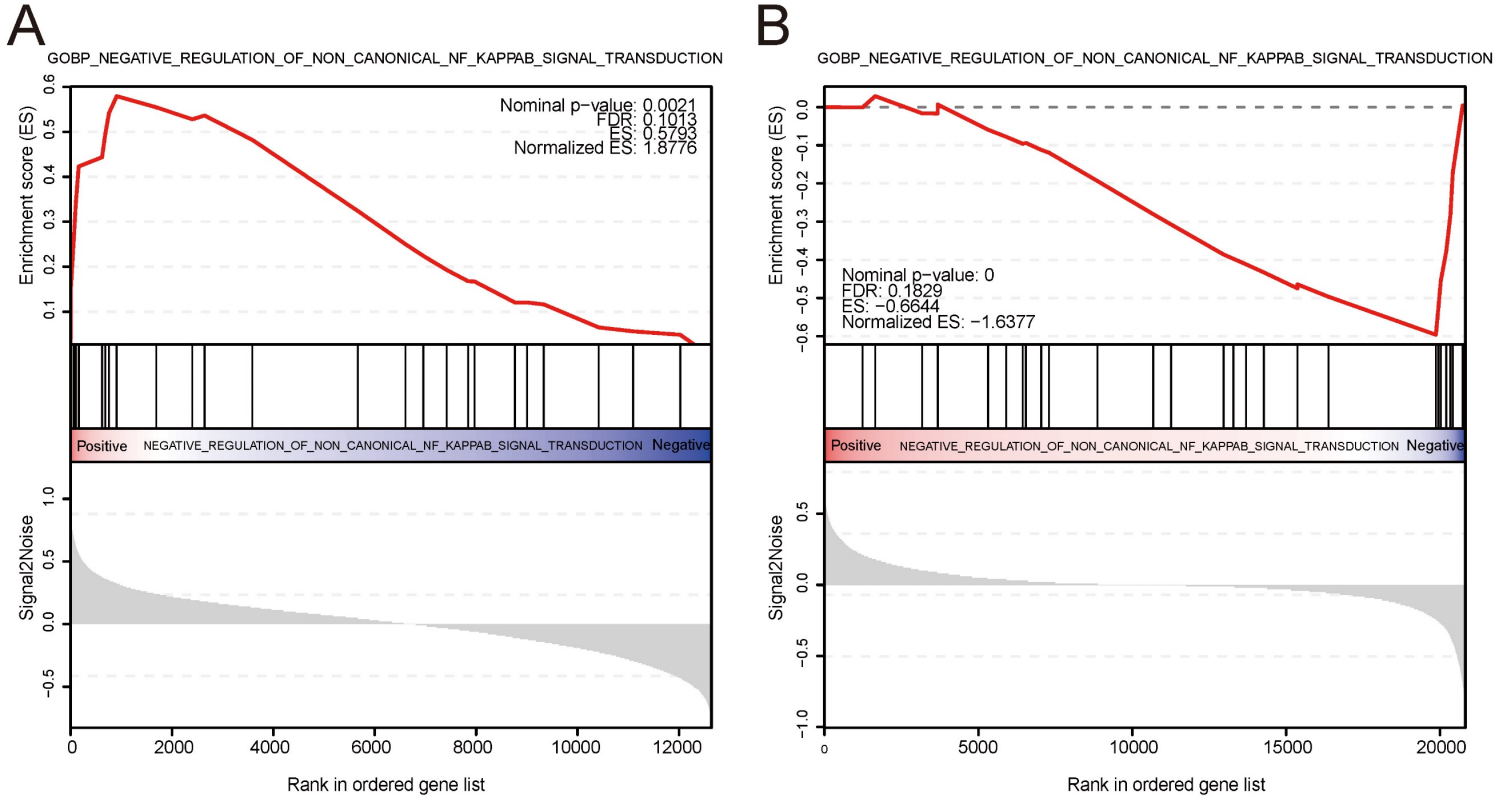
** ssGSEA.** (A) AQP9 was involved in the negative regulation of non classical NF-kappaB signaling pathway transduction in IBD, showing upregulation. (B) AQP9 was involved in the negative regulation of the non classical NF-kappaB signaling pathway in psoriasis, showing downregulation.

We conducted ssGSEA on the IBD and psoriasis training datasets based on the expression levels of the shared diagnostic gene AQP9 in the IBD and psoriasis samples, respectively. The findings indicated that AQP9 plays a role in negatively regulating the non-classical NF-kappaB signaling pathway in both two conditions, showing upregulation in IBD and downregulation in psoriasis. Figure 6 showed the visualization results of ssGSEA and provides ES, NES, FDR, and P values.

### Analysis of immune infiltration involving the shared diagnostic gene

**Figure 7:**
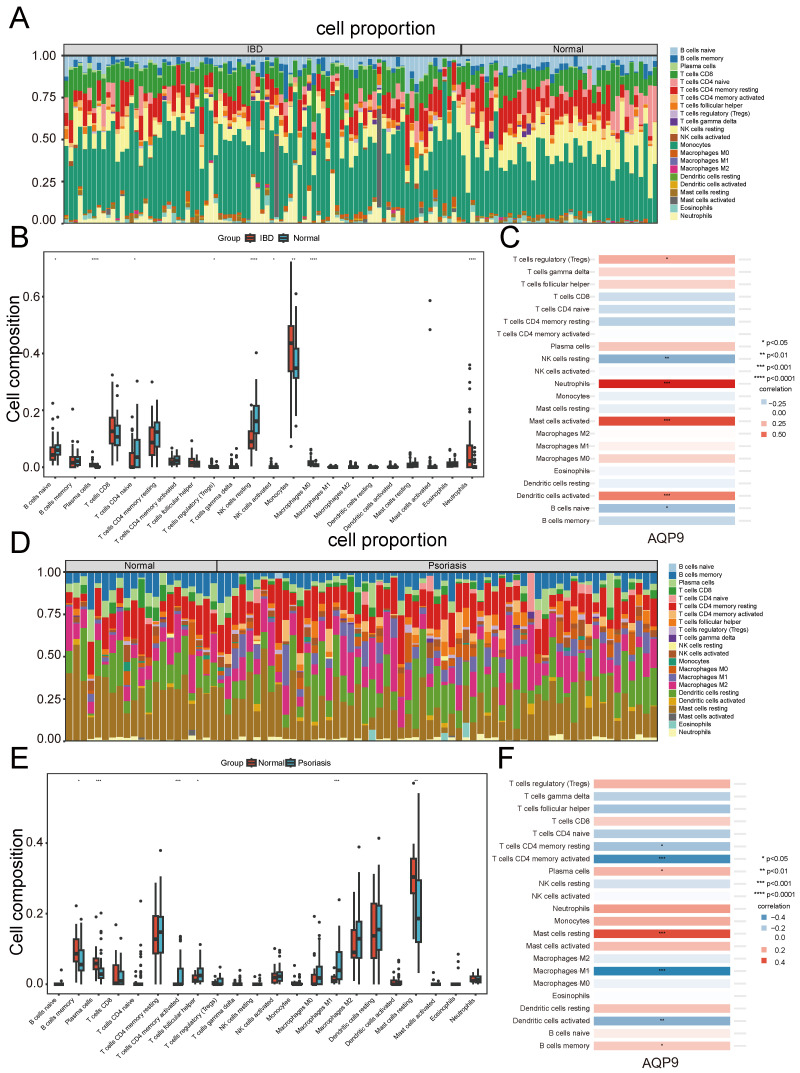
** Immune infiltration analysis.** (A) Immune cell stacking bar plot in the IBD group. (B) The box plot showed the differential composition of different types of immune cells between IBD samples and control samples. (C) The correlation between AQP9 expression and various types of immune cells in the IBD group. (D) Immune cell stacking bar plot in the psoriasis group. (E) The box plot showed the differential composition of different types of immune cells between psoriasis samples and control samples. (F) The correlation between AQP9 expression and various types of immune cells in the psoriasis group.

We chose the CIBERSORT method to analyze the presence of immune cells across different groups. A bar chart was used to depict the proportions of 22 immune cells in each group. Significant differences in the percentages of T cells, plasma cells, NK cells, monocytes, dendritic cells, and mast cells between IBD and psoriasis were clearly depicted in the bar chart (Figure 7A and Figure 7D). In the IBD samples, there was an increase in plasma cells, monocytes, Macrophages M0, and neutrophils, while T cells CD4 naive and NK cells resting were decreased compared to the control samples. (Figure 7B). In psoriasis samples, there was an increase in T cells CD4 memory activated, T cells follicular helper, and Macrophages M1, while a decrease was observed in B cells memory, plasma cells, and mast cells resting. (Figure 7E). Furthermore, we investigated the correlation between the shared diagnostic gene and immune cell content. In IBD samples, AQP9 showed significant positive correlations with neutrophils, mast cells activated, dendritic cells activated, and T cells regulatory (Tregs), while it had significant negative correlations with NK cells resting and B cells naive (Figure 7C). In psoriasis samples, AQP9 exhibited significant negative correlations with T cells CD4 memory activated, T cells CD4 memory resting, Macrophages M1, and dendritic cells activated, while it showed significant positive correlations with mast cells resting, plasma cells, and B cells memory (Figure 7F). Immune infiltration analysis indicated that immune responses involving T cells, macrophages, and dendritic cells play vital roles in the pathogenesis of IBD and psoriasis.

### Potential drug prediction

Based on the shared diagnostic gene AQP9, we conducted potential drug predictions with the Coremine database and the DsigDB on the Enrichr platform. We obtained information from the DsigDB that drugs such as securinine, ouabain, and mebendazole might have therapeutic effects on both two diseases (Table S3). At the same time, we obtained from the Coremine database that five traditional Chinese medicines, including Hua sang bai pi, Bai shu, Chi shao, Dan shen, and Feng ru, may have alleviated two diseases (Table S4).

### Experimental validation of AQP9 expression in colitis and psoriasis mice via RT-PCR

**Figure 8:**
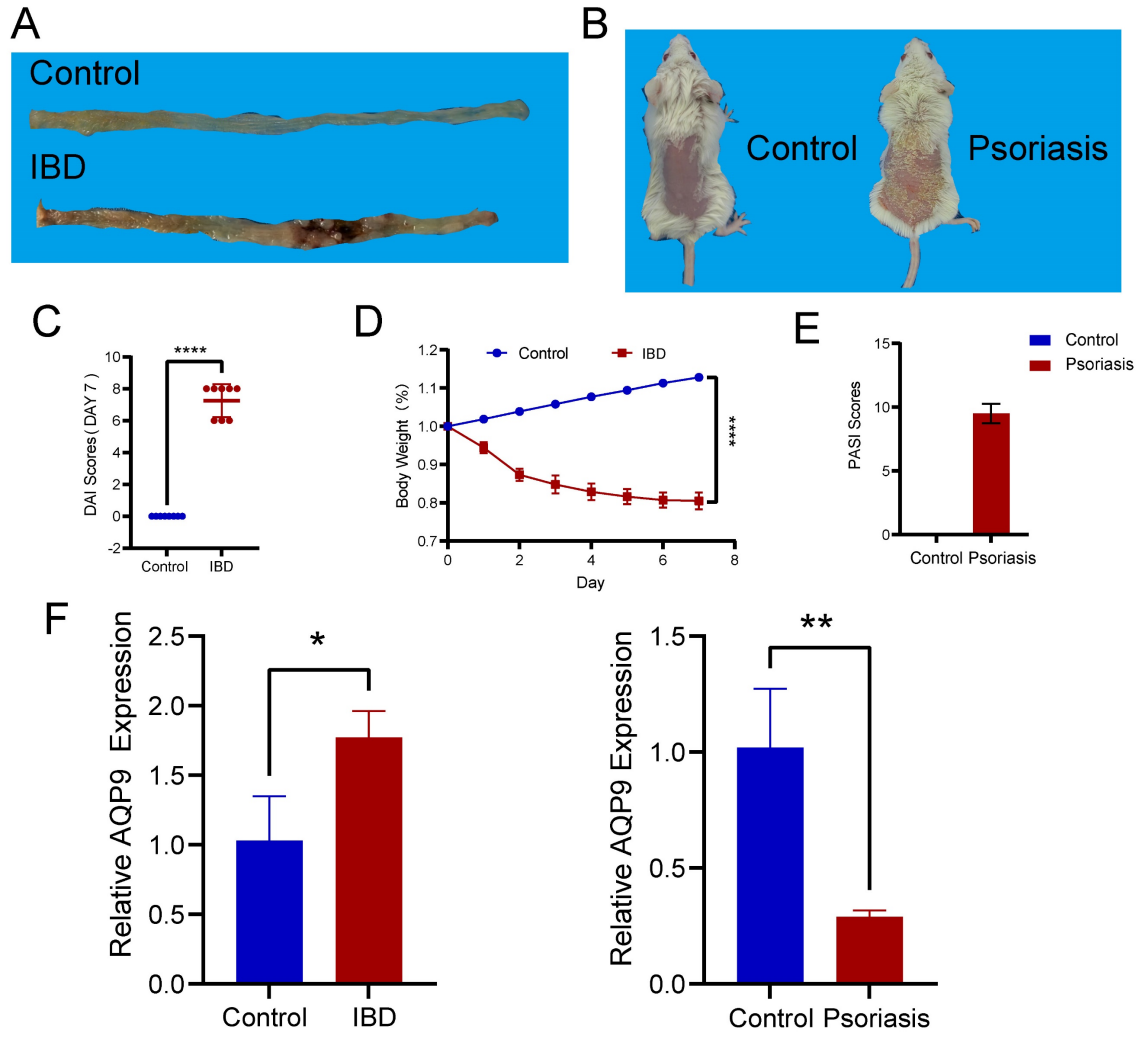
** Experimental assays of AQP9 expression in colitis and psoriasis mice via RT-PCR.** (A) Colon tissue in control and IBD group. (B) Skin tissue in control and psoriasis group. (C) DAI Scores. (D) Changes in body weight. (E) PASI Scores. (F) Expression levels of AQP9 in colitis and psoriasis mice compared with control group. *P< 0.05, **P<0.01.

We induced colitis in mice with TNBS and psoriasis with imiquimod (Figure 8A, B). To verify the effectiveness of the animal models, we conducted DAI scoring and monitored weight for the colitis mice (Figure 8C, D), while PASI scoring was performed on the skin lesions of the psoriasis mice (Figure 8E). Following this, we carried out RT-PCR on intestinal tissues from both normal and TNBS-induced colitis mice, as well as on skin tissues from healthy and imiquimod-induced psoriasis mice. Our experimental results validated the insights obtained from our bioinformatics analysis, indicating that AQP9 expression was elevated in the intestinal tissues of TNBS-induced colitis mice and decreased in the skin lesions of psoriasis mice (Figure 8F).

### Experimental validation of AQP9 expression in colitis and psoriasis mice via Western blot and IHC

**Figure 9:**
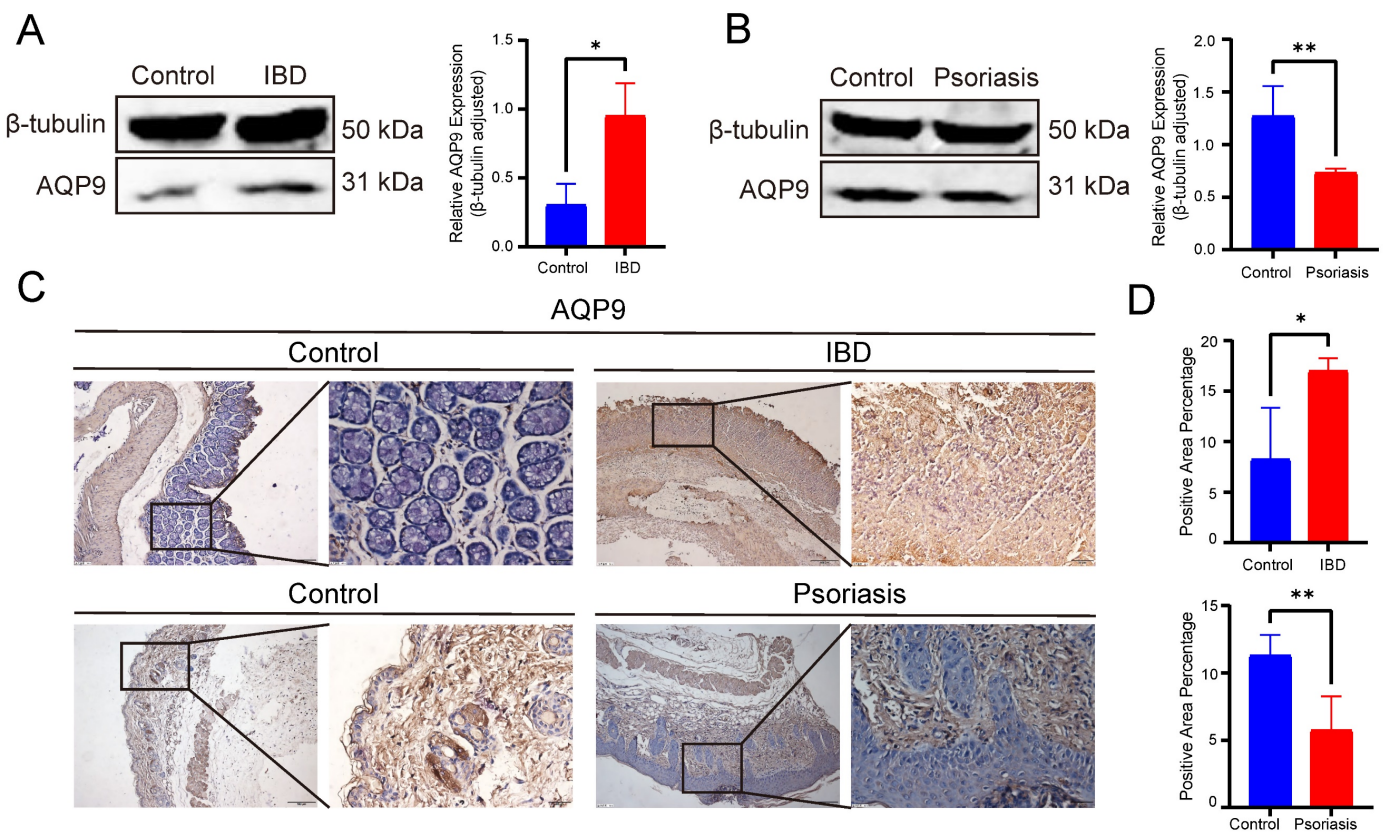
** Experimental assays of AQP9 expression in colitis and psoriasis mice via Western blot and IHC.** (A) Western blot analysis showed increased protein level of AQP9 in IBD compared to controls. β-tubulin served as loading control. (B) Western blot analysis showed decreased level in psoriasis compared to controls. β-tubulin served as loading control. (C) Representative immunohistochemistry images of AQP9 expression in IBD and psoriasis. (D) Comparison of immunohistochemistry staining area. *P< 0.05, **P<0.01.

Western blot analysis further confirmed the opposite expression patterns of AQP9 in IBD and psoriasis at the protein level. In comparison with the control group, AQP9 protein level was significantly elevated in the colonic tissues of TNBS-induced colitis mice, whereas it was markedly reduced in psoriatic skin lesions (Figure 9A-B). In order to further validate, we performed immunohistochemical analysis to examine the protein level of AQP9. In the colitis mice, AQP9 protein level was significantly higher compared to the control group. In contrast, AQP9 protein level was substantially lower in psoriatic skin lesions than in normal skin tissues (Figure 9C-D). These findings suggest that AQP9 exhibits distinct expression patterns in IBD and psoriasis, indicating its potential involvement in the differential regulation of inflammatory responses across the two diseases.

## Discussion

The comorbidity of IBD and psoriasis often interacts and contradicts each other in treatment. IBD patients with concomitant psoriasis have relatively poor treatment response compared to those without concomitant IBD, and the treatment effect of psoriasis patients with concomitant IBD is also poor. Meanwhile, IBD patients are prone to contradictory triggering of psoriasis when using biologics, and vice versa. Further research is needed to explore common pathogenesis and potential diagnostic and therapeutic targets. While both diseases involve genetic, environmental, and immune factors, their comorbidity mechanisms remain unclear. In this study, we first screened IBD and psoriasis datasets from GEO, conducted differential gene expression analysis and WGCNA, and identified AQP9 as a shared diagnostic gene using three machine learning methods. ROC curves tested its diagnostic efficacy. GSEA revealed AQP9's involvement in the negative regulation of non-classical NF-kappaB signaling, showing opposite trends in both diseases. Immune infiltration analysis found AQP9 expression significantly correlated with macrophages, T cells, and dendritic cells. Drug prediction identified potential treatments for both conditions. Experimental assays in TNBS-induced colitis and imiquimod-induced psoriasis mice confirmed AQP9 expression via RT-PCR, Western blot and IHC. In summary, AQP9 and the potential comorbidity mechanisms provide new directions for diagnosing and treating IBD and psoriasis.

Aquaporins (AQPs) are a group of membrane channel proteins that play a crucial role in facilitating water transport, energy regulation, and maintaining redox homeostasis across cell membranes 13, 14. Aquaporin-9 (AQP9) is part of the aquaporin family, facilitating the transport of water and small molecules like glycerol and hydrogen peroxide 15-17, playing a vital role in regulating osmotic pressure and energy metabolism 14. AQP9 is primarily found in liver cells and a variety of immune cells, including neutrophils, T cells, and macrophages 18-20. Recently, AQP9 had been found to be associated with inflammation 21, immune related diseases 14, and tumor related diseases 22-24.

In terms of involvement in inflammation, the study by Angela Tesse *et al.* demonstrated that AQP9 plays a role in the initial acute phase of lipopolysaccharide-induced endotoxin shock, implicating the NF-kappaB signaling pathway 25. Cheng *et al.*'s study showed that AQP9 could affect lipid metabolism in NAFLD, and knocking out AQP9 can decrease lipid toxicity, which subsequently lowers inflammation and oxidative stress, ultimately hindering the progression of NAFLD 26. In recent years, the significance of AQP9 within the immune system has increasingly come to light. AQP9 could modulate the movement of different types of immune cells and was regarded as one of the most significant aquaporins in the immune system 14. The importance of AQP9 expression in neutrophil migration had been thoroughly documented 27. In addition, AQP9 appeared to be involved in macrophage migration and in regulating CD8+T cell lifespan 28. Overall, AQP9 is intricately linked to inflammatory and immune related diseases.

The imbalance of aquaporins is associated with mucosal damage in several inflammatory diseases, including ulcerative colitis and Crohn's disease 29. Previous studies had shown that the mRNA and protein expression levels of AQP4 and AQP8 decreased in human colitis specimens and DSS induced mouse colitis specimens compared to normal controls, and changes in AQP mRNA and protein expression could be measured after 1 day of DSS induction, with almost no significant intestinal epithelial damage observed at this time point 30, 31. This suggested that AQPs might serve as biomarkers for early diagnosis of IBD. Another study showed that knocking out AQP3 could lead to impaired proliferation of intestinal epithelial cells. Mice with AQP3 gene knockout in DSS-induced colitis exhibited more severe colon pathology and a reduced survival rate compared to wild-type mice. Wild-type mice showed basal crypt cell proliferation after intestinal epithelial cell damage, while AQP3 knockout mice showed reduced epithelial cell proliferation 32. We had learned from the above research that AQPs were associated with the mucosal damage mechanism of IBD. At present, there had been many reports detailing the role of AQP9 in liver cancer, renal cell carcinoma, and NAFLD, but no reports had been found on the pathogenesis of AQP9 in the comorbidity of IBD and psoriasis. Moreover, the report of the role of AQPs in IBD had been issued for a long time, and the expression level is still controversial, requiring further verification. Through bioinformatics analysis and experimental assays, we learned that AQP9 expression levels were elevated in IBD and decreased in psoriasis, which offered new insights into the comorbidity mechanisms of IBD and psoriasis.

Aquaporins play a crucial role in skin barrier disruption in inflammatory skin diseases. AQPs are found in various cell types within the skin and contribute to essential functions, such as hydration, establishing water permeability barriers, and mediating immune responses 17. Previous studies had observed changes in the levels of AQPs in inflammatory skin disorders, including atopic dermatitis and psoriasis 33-35, and had developed new drug targets related to AQPs 36. Multiple research findings suggested AQP3 may play a role in the development of psoriasis 17, 37, 38. AQP9 is a homolog of AQP3, yet in one study 39, it was shown that retinoic acid stimulation could increase AQP3 expression and decrease AQP9 expression, indicating that the regulatory mechanisms of AQP9 in epidermal keratinocytes may differ from those of AQP3. AQP9 can regulate the balance of water and glycerol in the epidermis. In summary, AQP9 might also participate in the mechanism of epidermal damage in psoriasis by regulating the water glycerol balance of the epidermis and the migration and polarization of immune cells.

The NF-kappaB pathway was a common signaling pathway in inflammatory and immune diseases, and could be mainly divided into two types: the canonical and the non-canonical NF-kappaB signalling pathways 40, 41. The NF-kappaB pathway had been reported in previous studies to be associated with inflammation and metabolic processes related to IBD 42, 43 and psoriasis 44, 45, and had become a potential target for drug therapy 43, 46. The involvement of AQPs in the regulation of the NF-kappaB pathway during disease processes had been reported in diseases such as brain edema 47 and lung injury 48. The report of the regulatory role of AQPs in the NF-kappaB pathway in IBD had also been published. In the study by Liuhua Wang *et al.*, knocking out the AQP4 gene could inhibit the activation of NF-kappaB, inhibit the secretion of pro-inflammatory cytokines, reduce the influx of inflammatory cells into the colon mucosa, and thus enhance the tolerance of mice to DSS induced experimental colitis 49.

At present, there had been no relevant research on the comorbidity mechanism between AQP9, NF-kappaB pathway, IBD, and psoriasis. In our research, bioinformatics were applied to identify the shared diagnostic gene AQP9. Previous studies indicate that: IBD was a chronic non-specific inflammation of the intestine; psoriasis was a chronic inflammation of the epidermis; and both diseases involve the regulation of water and glycerol balance in epithelial cells. Dysregulation of aquaporins can lead to fluid imbalance, which in turn can lead to diseases. AQP9, a member of AQPs, is a water glycerol channel protein known to be expressed in both human and animal intestinal epithelial cells and epidermal cells. Previous studies have linked the NF-kappaB pathway to the disease processes of IBD 42, 43 and psoriasis 44, 45. Research on AQP4, a member of AQPs, regulating the inflammatory state of IBD through the NF-kappaB pathway had also been reported. Moreover, AQP9 had been shown to be associated with macrophage polarization 50, 51, participating in T cell differentiation 28 and neutrophil migration. Our research findings on immune infiltration analysis were consistent with previous research, showing significant differences in percentage of immune cells, including neutrophils, T cells, and macrophages between the disease group and the normal group in the two diseases.

Based on the above findings, we hypothesized that AQP9, as a water glycerol channel protein, might participate in the comorbidity mechanism of IBD and psoriasis by regulating the water glycerol balance of intestinal and epidermal epithelial cells, and ssGSEA showed that AQP9 may play a role in regulating the NF-kappaB signaling pathway in both diseases. Immunoinfiltration analysis and previous studies had also shown that AQP9 could impact the process of the diseases by regulating the differentiation and migration of immune cells, including neutrophils, T cells, and macrophages. It may also reverse the imbalance of water and glycerol regulation in epithelial cells, further exacerbating the progression of the original condition after the epithelial barrier of IBD and psoriasis patients was damaged. Based on previous studies on drug targets of AQPs 18, AQP9 might become a new predictive factor and potential therapeutic target in the comorbidity of IBD and psoriasis.

Although we had identified shared diagnostic gene for IBD and psoriasis through bioinformatics methods, it was not clear the specific comorbidity mechanism of AQP9, and the developmental sequence of the alteration of the expression of AQP9 was unclear whether it is prior to the occurrence of the two inflammatory diseases or secondary to the two inflammatory diseases, as well as whether AQP9 was involved in the NF- kappaB pathway in these two diseases and the regulatory mechanism thereof were still unclear, so further research still requires rigorous experimental verification. Additionally, research indicated that knocking out AQP9 results in weight loss in mice due to alterations in glycerol metabolism. This leads to liver cell death and the infiltration of inflammatory cells. Furthermore, the absence of AQP9 might initiate new immune and inflammatory responses, causing scattered and mild necrosis of liver cells and compensatory proliferation of liver cells 20, which suggested that changes in AQP9 expression might lead to other adverse reactions in the body. Moreover, there were currently few inhibitors targeting AQP9, which contained highly toxic substances such as mercuric chloride. This might be a limitation in the research of AQP9 as a therapeutic target, and further exploration was still needed in the future.

In conclusion, we identified AQP9 as a potential predictive biomarker for IBD and psoriasis. Meanwhile, AQP9 may regulate the differentiation and migration of immune cells, including neutrophils, T cells, and macrophages. In summary, this study provided a potential predictive biomarker and pathogenesis for the comorbidity of IBD and psoriasis.

## Supplementary Material

Supplementary figures and tables.

## Figures and Tables

**Table 1:** Shared genes for UC and psoriasis.

Intersection	Genes
UC	CYP1B1, AMMECR1, IL1R2, AQP9, VAMP1, PRSS53, FCF1, ZNF14
Psoriasis	ABCA8, IL1R2, AQP9, ZNF14, FBXO42, ZWILCH, EGR1

**Table 2:** Shared genes for CD and psoriasis.

Intersection	Genes
CD	DLG5, CYP1B1, MMP9, IL1R2, AQP9, ZBTB16, SOCS3, H2BC5, GZMB, TUBA4A, LCN2, H2AC6, BEX3
Psoriasis	KLF6, SLC2A3, ATF3, AMMECR1, AQP9, JMJD6, H2BC5

**Table 3:** AUC values of shared diagnostic genes in validation datasets.

	Internal validation					External validation			
Disease	IL1R2	AQP9	ZNF14	H2BC5		IL1R2	AQP9	ZNF14	H2BC5
UC	83.7	93.681	83.974	—		65.933	90.394	50.914	—
CD	—	89.629	—	93.543		—	93.909	—	61.333
Psoriasis	78.728	78.689	92.194	80.562		75.641	82.906	93.162	51.709

## Abbreviations

IBD: Inflammatory bowel disease
CD: Crohn's disease
UC: Ulcerative colitis
GEO: Gene Expression Omnibus
WGCNA: Weighted Gene Co-expression Network Analysis
KEGG: Kyoto Encyclopedia of Genes and Genomes
GO: Gene Ontology
ROC: Receiver Operating Characteristic
AUC: Area Under Curve
SD: standard deviation
ssGSEA: Single sample gene set enrichment analysis
EIMs: Extraintestinal manifestations
DEGs: Differentially Expressed Genes
LASSO: Least Absolute Shrinkage and Selection Operator
SVM-RFE: Support Vector Machine Recursive Feature Elimination
RF: Random Forest
DsigDB: Drug Signature Database
AQP9: Aquaporin-9
AQPs: Aquaporins
TNBS: 2,4,6-Trinitrobenzenesulfonic acid
RT-PCR: Real-time polymerase chain reaction
IHC: Immunohistochemistry
SDS-PAGE: Sodium dodecyl sulfate-polyacrylamide gel electrophoresis
PVDF: Polyvinylidene difluoride
