# Supplementary material for: Exploring the Shared Diagnostic Genes in IBD and Psoriasis through Bioinformatics and Experimental Assays
Source: Int J Med Sci. 2025 Mar 3;22(7):1680–97. doi: 10.7150/ijms.107018 (PMC11905276; doi:10.7150/ijms.107018)
Supplement: Supplementary file 1 — Supplementary figures and tables. [file ijmsv22p1680s1.pdf]

Supplementary Materials

List of Supplementary Materials

Figure S1 to S2

Table S1 to S4

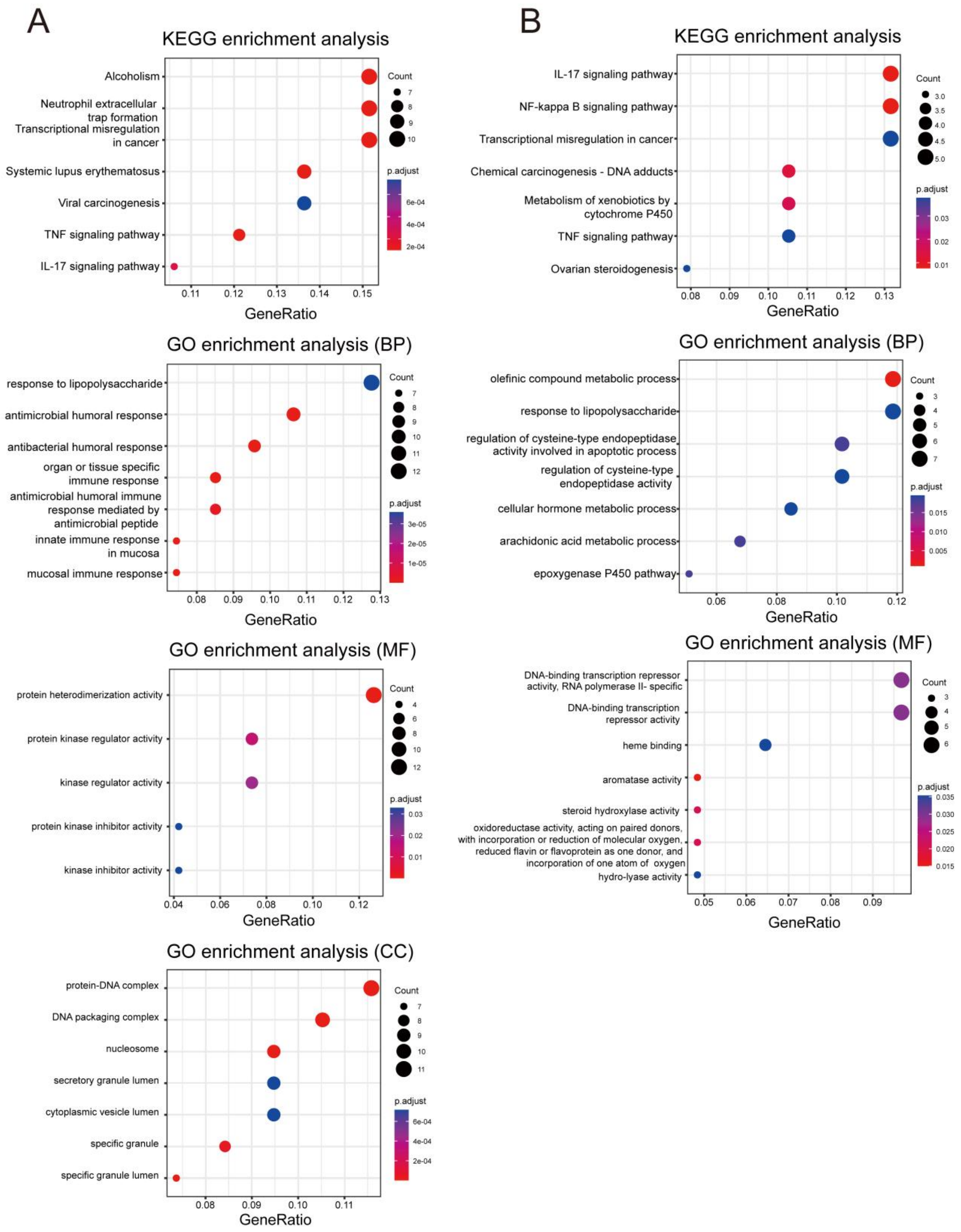

Figure S1. Functional enrichment analysis. (A) The enrichment results for UC-psoriasis shared genes. (B) The enrichment results for CD-psoriasis shared genes.

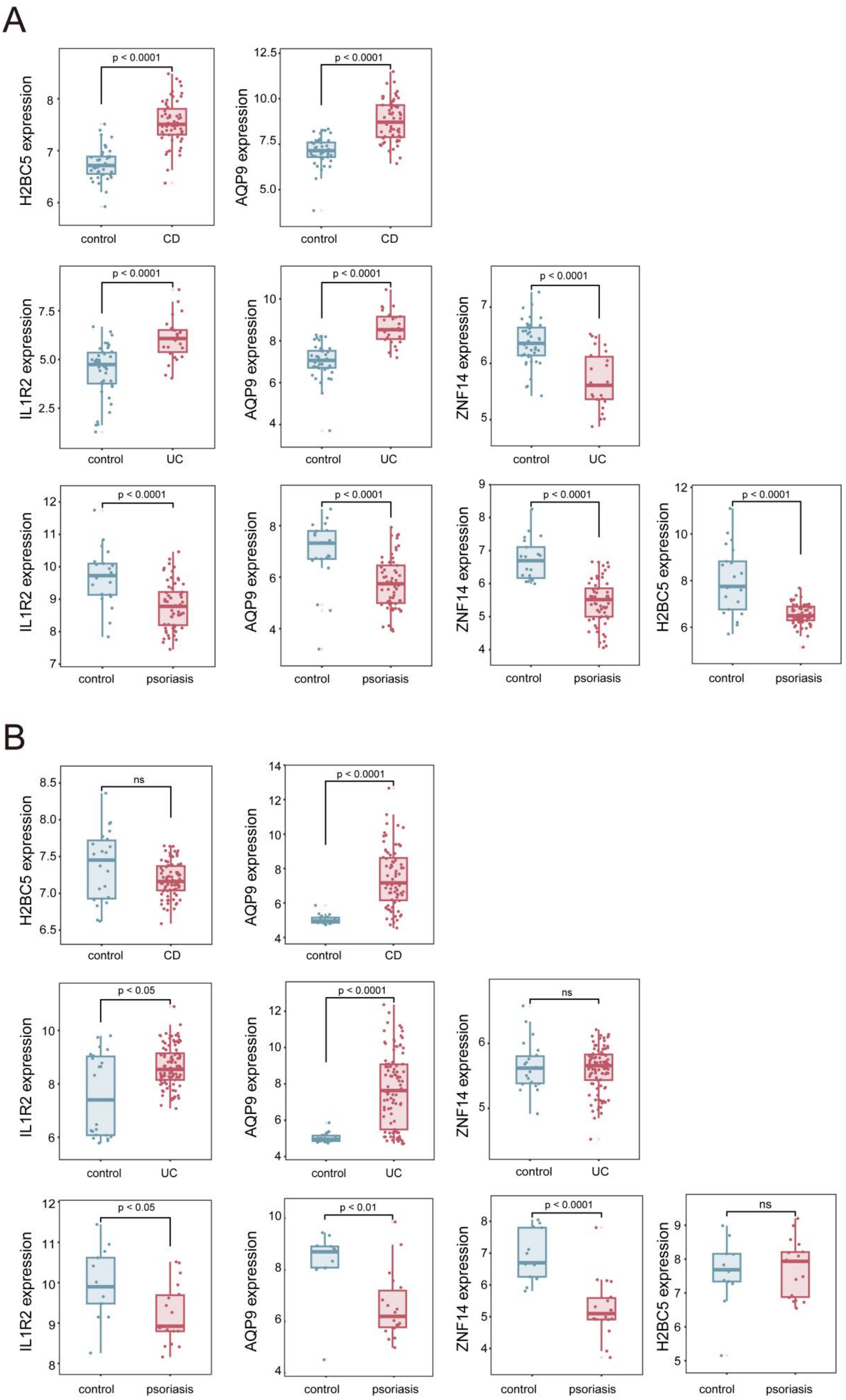

Figure S2. Expression trends of shared genes. (A) The expression trends of shared genes in the internal validation. (B) The expression trends of shared genes in the external validation.

Table S1. Primer sequences for Real-Time PCR.

Gene		Primer sequences
GAPDH	Forward	5′-AGGTTGTCTCCTGCGACTTCA-3′
	Reverse	5′-GGGTGGTCCAGGGTTTCTTA-3′
AQP9	Forward	5′-GCTCAAGCAGTCCTCAGTCG-3′
	Reverse	5′-GACACCGCTGGGTTGATGTG-3′

Table S2. Shared genes details between UC/CD and psoriasis.

Intersection	Share Genes
UC and Psoriasis	CLUHP3, APOBEC3B, ZWILCH, E2F8, KIF11, AMMECR1, CYP2E1, BCL2A1, CLEC7A, VNN1, XAF1, PRSS53, SMOX, CRCT1, MMP9, CXCL1, KLF6, ADM, GADD45G, EGR1, ZNF14, PMAIP1, GPRC5A, FOSB, THBS1, ZBTB20, H2AC6, BHLHE41, CLSTN2, ABLIM3, FGF2, CYP1B1, AQP9, ABCA8, PTGS2, FKBP5, CYP1A1, ZBTB16, ADH1B, SELENBP1, MEG3, SREBF2, NFKBIA, LIG1, ENOSF1, IL1R2, CIT, MICOS10P1, FCF1, ZFAND3, HPS6, METTL17, RPS6KA3, ALS2CL, LOXL1, COG5, ZNF443, B4GALT3, MAZ, VAMP1, ZC3H3, CCPG1, FBXO42, ETV3
CD and Psoriasis	PLAAT1, ARG1, NDC80, AMMECR1, CCNA2, SLPI, BCL2A1, CX3CR1, PI3, VNN1, IFI44L, XAF1, LCN2, SMOX, MMP9, GZMB, PDZK1IP1, CXCL1, EIF5A, LTF, ATF3, KLF6, TSC22D1, SPRY2, PPP1R15A, EGR1, GPRC5A, HEY1, H2BC5, FOSB, H2BC21, IL6, CLDN5, IER3, THBS1, SOCS3, H2AC8, H2AC6, ZNF571, SLC13A2, CLSTN2, CAVIN2, ABLIM3, CYP1B1, RAI2, AQP9, GRB14, PTGS2, CYP1A1, ZBTB16, SELENBP1, G0S2, SRSF2, ZFP36, CDKN1A, SLC2A3, ICAM1, ADM, NFIL3, PDE4B, IL1R2, PTX3, GADD45B, H2BC7, H2BC10, H2BC6, NEU1, CSF1, VEGFA, TUBA4A, JMJD6, HLX, GLUL, H2BC8, QARS1, MGAT4B, MBTPS2, H2BC4, GAS2L1, MAFF, DUSP1, CTTN, TUBA1B, BTG2, ETS2, MARCKS, DLG5, MLH3, ARL4A, FOS, DDIT3, TIPARP, BEX3, CCPG1, S1PR5

Table S3. Prediction of potential therapeutic drugs from DsigDB.

Term	P-value	Adjusted P-value	Combined Score
securinine HL60 UP	0.002199961	0.017599686	122117.0656
ANTIMONY CTD 00005424	0.003949942	0.020799726	110243.8958
Dichloromercury CTD 00006273	0.004449938	0.020799726	107815.3803
cholecalciferol CTD 00005655	0.005199931	0.020799726	104635.2494
COBALT CTD 00005696	0.008649907	0.029656824	94182.34859
mebendazole HL60 UP	0.017299864	0.045479659	79737.39093
3'-Azido-3'-deoxythymidine CTD 00007047	0.018699859	0.045479659	78096.55061
ouabain HL60 UP	0.018949858	0.045479659	77816.07855
digitoxigenin HL60 UP	0.02104985	0.045926946	75591.8137

Table S4. Prediction of potential therapeutic drugs from Coremine database.

Gene	Association Name	Association Type	Significance
AQP9	hua sang bai pi	Traditional chinese medicine	0.000836203
AQP9	bai shu	Traditional chinese medicine	0.009002914
AQP9	chi shao	Traditional chinese medicine	0.019034745
AQP9	dan shen	Traditional chinese medicine	0.01991576
AQP9	feng ru	Traditional chinese medicine	0.025151473
